# In-field High Throughput Phenotyping and Cotton Plant Growth Analysis Using LiDAR

**DOI:** 10.3389/fpls.2018.00016

**Published:** 2018-01-22

**Authors:** Shangpeng Sun, Changying Li, Andrew H. Paterson, Yu Jiang, Rui Xu, Jon S. Robertson, John L. Snider, Peng W. Chee

**Affiliations:** ^1^School of Electrical and Computer Engineering, College of Engineering, University of Georgia, Athens, GA, United States; ^2^Department of Crop and Soil Sciences, College of Agricultural and Environmental Sciences, University of Georgia, Athens, GA, United States; ^3^Department of Genetics, Franklin College of Arts and Sciences, University of Georgia, Athens, GA, United States

**Keywords:** field-based high throughput phenotyping, 3D point cloud, morphologic traits, plant growth analysis, LiDAR

## Abstract

Plant breeding programs and a wide range of plant science applications would greatly benefit from the development of in-field high throughput phenotyping technologies. In this study, a terrestrial LiDAR-based high throughput phenotyping system was developed. A 2D LiDAR was applied to scan plants from overhead in the field, and an RTK-GPS was used to provide spatial coordinates. Precise 3D models of scanned plants were reconstructed based on the LiDAR and RTK-GPS data. The ground plane of the 3D model was separated by RANSAC algorithm and a Euclidean clustering algorithm was applied to remove noise generated by weeds. After that, clean 3D surface models of cotton plants were obtained, from which three plot-level morphologic traits including canopy height, projected canopy area, and plant volume were derived. Canopy height ranging from 85th percentile to the maximum height were computed based on the histogram of the z coordinate for all measured points; projected canopy area was derived by projecting all points on a ground plane; and a Trapezoidal rule based algorithm was proposed to estimate plant volume. Results of validation experiments showed good agreement between LiDAR measurements and manual measurements for maximum canopy height, projected canopy area, and plant volume, with *R*^2^-values of 0.97, 0.97, and 0.98, respectively. The developed system was used to scan the whole field repeatedly over the period from 43 to 109 days after planting. Growth trends and growth rate curves for all three derived morphologic traits were established over the monitoring period for each cultivar. Overall, four different cultivars showed similar growth trends and growth rate patterns. Each cultivar continued to grow until ~88 days after planting, and from then on varied little. However, the actual values were cultivar specific. Correlation analysis between morphologic traits and final yield was conducted over the monitoring period. When considering each cultivar individually, the three traits showed the best correlations with final yield during the period between around 67 and 109 days after planting, with maximum *R*^2^-values of up to 0.84, 0.88, and 0.85, respectively. The developed system demonstrated relatively high throughput data collection and analysis.

## Introduction

The global population is estimated to approach nine billion by 2050, and demand for food and fiber crops is expected to increase by 60% (Tilman et al., 2011; Gerland et al., 2014). New plant breeding approaches need to be developed to overcome these tremendous challenges. An important step in this direction is to gain a better understanding of the relationship between genotype and phenotype (Goggin et al., 2015; Großkinsky et al., 2015; Rahaman et al., 2015). However, in-field high throughput phenotyping technologies, which can facilitate automatic measurement of phenotypic traits over the entire growing season, are still considered to be a major bottleneck limiting crop improvement (Furbank and Tester, 2011; Cobb et al., 2013).

Plant morphologic traits can often be used for evaluating plant growth (Hosoi and Omasa, 2012; Taheriazad et al., 2016), which determines plant performance in terms of final crop biomass and yield (Dhondt et al., 2013). Several studies showed that morphologic traits such as canopy height and leaf area index (LAI) were strongly related to plant species, type of cultivation, plant growth rate, and final yield (Gebbers et al., 2011; Sharma and Ritchie, 2015; Friedli et al., 2016; Sun et al., 2017). Importantly, plant growth and yield is dependent upon leaf area development, the average photosynthetic efficiency of all leaves in the plant canopy (Gardner, 1985; Krieg and Sung, 1986), and partitioning of dry matter to the harvested portion of the crop (Earl and Davis, 2003). Thus, plant canopy development should provide some indication of the crop's capacity for growth and yield.

The traditional manual measurement of plant morphologic traits is time consuming, labor intensive, and sometimes destructive. Novel technologies for plant phenotyping in a non-invasive and high throughput manner with high spatial and temporal resolution offer improved efficiency (Furbank and Tester, 2011; Dhondt et al., 2013; Großkinsky et al., 2015). Over the past decade, several non-invasive approaches using sensing technologies were developed for in-field phenotyping (Lin, 2015; Simko et al., 2016). Computer vision was one commonly-used technology. Usually, plant traits were extracted from color (RGB) images. Li et al. (2016) introduced a method for in-field cotton boll detection based on color and texture features using 2D color images. Si et al. (2015) developed a machine vision system to automatically recognize and locate apples; over 89.5% accuracy was achieved. Such 2D image based methods provide potential to conduct phenotypic measurements with a high spatial resolution, but are limited by plant occlusion. In addition, one major challenge with 2D digital image methods is that image quality is significantly affected by highly variable illumination conditions in the field, which limits automatic data processing (Li et al., 2014).

The use of 3D model-based methods for plant phenotyping are receiving increasing attention, as they permit multiple morphologic traits such as canopy height, plant volume, and LAI to be simultaneously extracted (Bietresato et al., 2016; Vazquez-Arellano et al., 2016; Gibbs et al., 2017) while mitigating plant occlusion. Moreover, 3D models have the potential to assist growers to continuously monitor and quantify plant growth and development, as well as plant responses to environmental stresses. A stereo-imaging based 3D reconstruction system was established to capture rape seedling leaf area and plant height (Xiong et al., 2017); two identical RGB cameras were utilized as an imaging unit. The mean error for leaf area and plant height measurements was 3.68 and 6.18%, respectively. The system was put in a well-designed box, in which homogenous illumination was provided. Thuy Tuong et al. (2015) developed a 3D reconstruction system based on 10 digital color cameras that were mounted on a custom structure, and an illumination system was used to enhance the visual texture of plants from all camera viewpoints. The system produced very high quality, dense, and complete point clouds. However, as both systems were designed for indoor use, they would need to be modified for field applications under natural illumination. 3D models can also be produced by time of flight (TOF) cameras; however, similarly to RGB image based methods, data quality would be significantly affected by sunlight under field conditions, which limits in-field applications. In Busemeyer et al. (2013) and Jiang et al. (2016) the TOF cameras were mounted inside an enclosure in order to mitigate the influence of sunlight.

Light detection and ranging (LiDAR) technology provides an alternative approach for 3D plant model reconstruction. LiDAR is a remote sensing technology to measure the distance between the sensor and an object of interest by illuminating the object with a laser and analyzing the TOF. LiDAR may be the best known and most widely used sensor for 3D canopy reconstruction (Deery et al., 2014; Gibbs et al., 2017). A 2D LiDAR collects two dimensional scans in a measured plane, and a 3D model can be obtained by moving the sensor along the perpendicular direction to the scanning plane. Although the spatial resolution of the 3D model produced by LiDAR is not as dense as those obtained by camera-based methods, it is sufficient for the extraction of most plant morphologic traits (Rosell-Polo et al., 2009; Bietresato et al., 2016; Sun et al., 2017). In Deery et al. (2014), a LiDAR (LMS400, SICK AG, Waldkirch, Germany) with a monochromatic red laser light source was used to generate intensity images of several crops including rice, wheat, and maize. It was concluded that LiDAR is a potential alternative to image-based methods for phenotyping morphologic traits at the plot or plant level under field conditions. Moreover, in contrast to image based methods, the LiDAR based method uses its own light source, mitigating problems with highly variable illumination conditions in the field. In addition, LiDAR can be used with a high scanning frequency and a large scanning range (Lin, 2015). Therefore, LiDAR has excellent potential for in-field plant phenotyping. 3D point clouds can also be generated by some other sensors such as triangulation line scanner (Paulus et al., 2014b) and ultrasonic sensing (Llorens et al., 2011). A very dense 3D model can be reconstructed using a triangulation line scanner and morphologic traits at the organ level could be extracted, however the relatively short working distance limits its application for large plants such as cotton.

Growth dynamics of plant morphologic traits provide important information toward determining plant productivity. Tessmer et al. (2013) described a high-throughput phenotyping platform for plant growth modeling and functional analysis (HPGA). Plant growth curves were generated by the platform, which were used to gain a deeper understanding of energy distribution. Friedli et al. (2016) introduced a terrestrial 3D laser scanner-based plant growth monitoring system which could be used for monitoring canopy height growth for different crops under field conditions. Paulus et al. (2014b) monitored the organ-specific growth dynamics of cereal plants with a high precision triangulation line laser scanner by scanning every 2–3 days. Three morphologic traits including leaf area, stem height, and plant volume were measured, allowing quantification of the growth dynamics of the barley plant.

In the present study, three morphologic traits—canopy height, projected canopy area, and plant volume—of cotton plants were derived based on data collected by a 2D LiDAR. Morphologic trait data was collected and growth analysis was conducted. Several improvements were made over previously mentioned studies. A 1-cm accuracy level RTK-GPS was used in this study in order to provide accurate spatial coordinates for LiDAR scans so that a precise 3D surface model could be reconstructed, which is important because the model is the basic dataset for further analysis. In addition, analyses of growth dynamics and correlation of morphologic traits with final yield were conducted over the growing season. The system repeatedly scanned plants over the growing season, permitting analysis of the effects of different cultivars on the growth and final yield of cotton plants.

The overall goal of this work was to develop a high throughput phenotyping system for morphologic traits of cotton plants using LiDAR under field conditions. This LiDAR-based system is one component of our broader effort to develop field-based high throughput phenotyping (HTP) systems, and it complements other image-based sensors such as Kinect V2, thermal camera, and hyperspectral camera. This LiDAR-based system provides accurate morphologic traits in a robust and fast way, and saves storage space and computing resources compared to image-based sensors. Specific objectives were to: (1) develop algorithms to extract multiple morphologic traits—canopy height, projected canopy area, and plant volume—from a 3D point cloud obtained with a 2D LiDAR; (2) conduct 4D monitoring (3D plant reconstructions over time) of the derived plant morphologic traits to detect growth patterns of plants from different cultivars; and (3) explore relationships between derived morphologic traits and final yield.

## Materials and methods

### Experimental field

**Figure 1:**
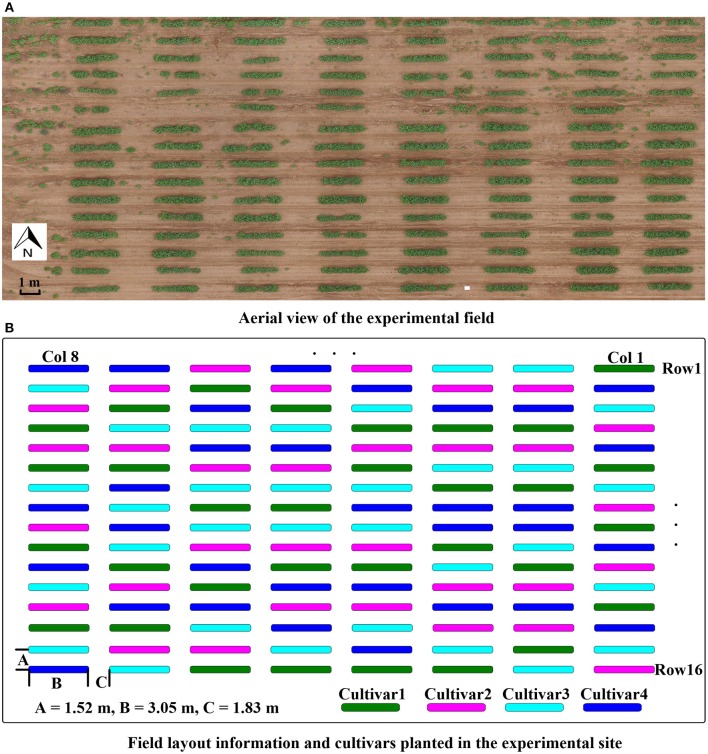
Experimental field layout. **(A)** Aerial view of the experimental field; **(B)** illustration of experimental design with cultivar and field layout information.

The study site was located at the Iron Horse Farm (IHF) in Greene County, GA, USA. The entire study included 128 plots arranged in 16 rows and 8 columns (Figure 1A), using a randomized complete block design with four cultivars of cotton and 32 replicate plots per cultivar. Four plots of each cultivar were planted in each column. The distribution of cultivars in each column was randomly assigned (Figure 1B). Plots were 3.05 m wide. A total of 15 seeds were sowed in each plot at spacing of 0.15 m. Inter-row spacing was 1.52 m, and inter-column spacing was 1.83 m. Cotton seeds were sowed on June 13, 2016.

The four cotton cultivars were GA2011158 (cultivar 1), GA2009037 (cultivar 2), GA2010074 (cultivar 3), and UA48 (cultivar 4) which is commercialized by the private seed company Americot. All four cultivars were developed by conventional breeding possessing no transgenic insects or herbicides tolerant traits. However, they have different fiber quality, growth habits, and plant architecture due to adaptation to different production regions. Cultivars 1, 2, and 3 are elite breeding lines developed by the University of Georgia cotton breeding program for adaptation to the southeastern cotton production region, bred to have indeterminate growth habit to take advantage of the long growing season in the southern US cotton belt. Plants from these cultivars will continue adding vegetative growth at the same time as the reproductive development, therefore they can grow excessively tall and rank in high nitrogen environment or if there are severe insects damage causing excessive square loss. Cultivar 4, on the other hand, was released by the University of Arkansas cotton breeding program, bred to have an early maturity growth habit for adaptation to the northern region of the US cotton belt. It has a determinate growth habit which resulting in shorter statured, extended sympodial branches, and shorter flowering date.

### Data acquisition

**Figure 2:**
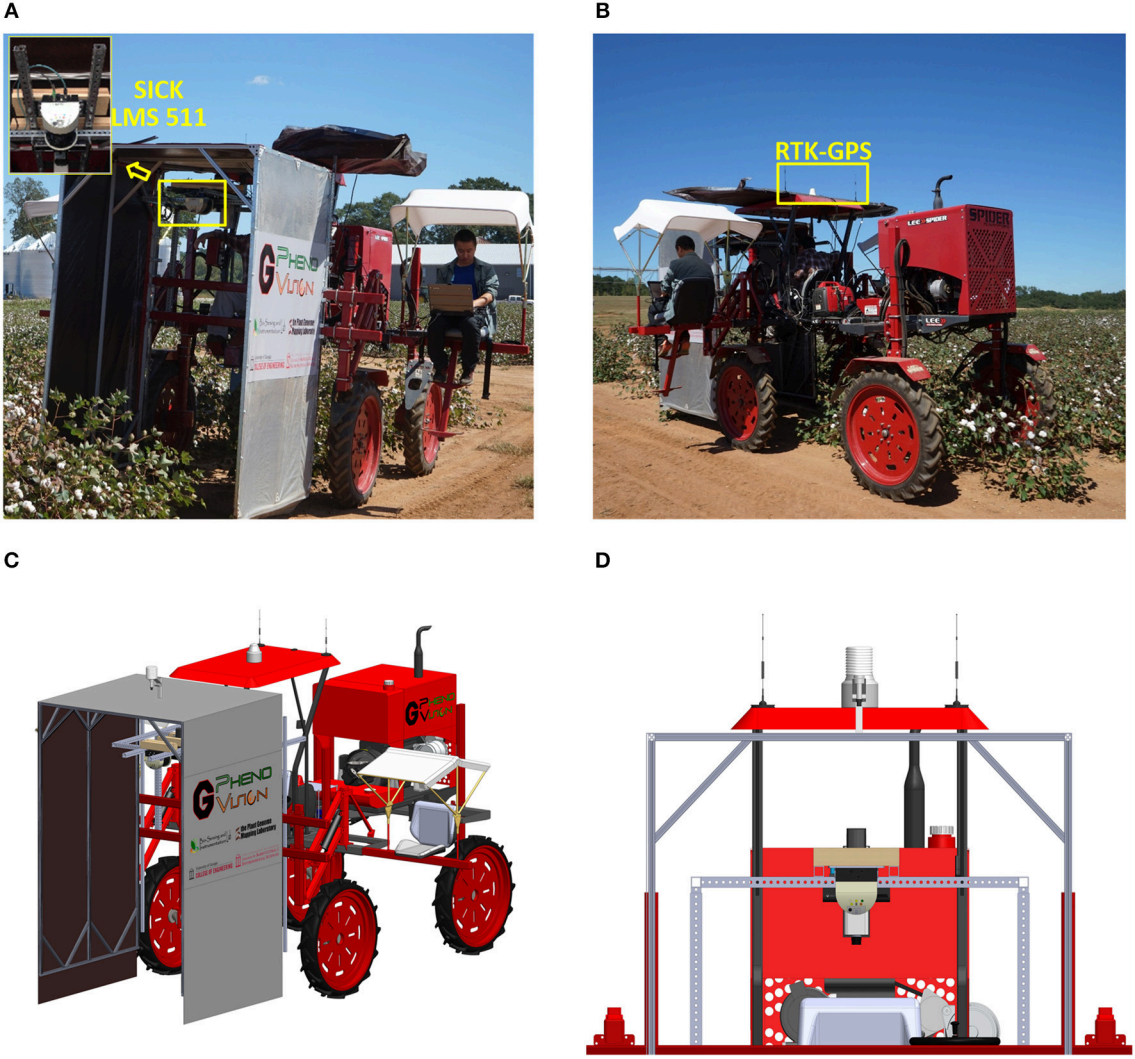
Data collection platform. **(A)** Front view; **(B)** back view; **(C)** 3D model of data collection platform; **(D)** zoomed view of sensors. The consent obtained from the depicted individual for the publication of these images was both informed and written.

The data collection system mainly consisted of a LiDAR (LMS 511 PRO SR, SICK AG, Waldkrich, Germany) (Figure 2A), an RTK-GPS (Cruizer II, Raven Industries Inc., Sioux Falls, SD, USA) (Figure 2B), and a rugged laptop as a DAQ (data acquisition) and storage device. The LiDAR was mounted on a tractor (Spider DL, LeeAgra, Inc., Lubbock, TX, USA) platform at a height of 2.4 m to scan cotton plots from directly above (Figure 2C). The RTK-GPS was mounted on the roof of the tractor, which provided the spatial coordinates during data collection. The LiDAR and RTK-GPS receiver were aligned to the center of the tractor (Figure 2D).

The LiDAR was developed for outdoor use, and measured in 2D radial coordinates from −5 to 185° with a maximum range of 80 m. Line scans could be acquired at a rate of 25–100 Hz with an angular resolution of 0.1667–1°. The built-in filters eliminated interference from particles of dust, raindrops, and snowflakes. An enclosure was used to provide a controlled environment for data acquisition. The RTK-GPS provided coordinates with 1 cm accuracy with an update rate up to 10 Hz.

Data collections were conducted in the field from July 26 to September 30, 2016, i.e., from 43 to 109 days after planting (DAP) (Table 1). When scanning plants, the angular resolution of the LiDAR was configured to be 0.33° with a sampling frequency of 50 Hz, and the echo filter and particle filter were enabled. The update frequency of the RTK-GPS was 5 Hz. The tractor scanned the field row by row. For each row, the tractor traveled from column 1 to column 8 with an average speed of about 0.5 m/s. Since the first two sample dates—July 26 and 28—were close to each other, the data for July 26 were not presented except for growth rate analysis.

**Table 1:** Summary of data collection dates (Year: 2016).

**Period**	**P1**	**P2**	**P3**	**P4**	**P5**	**P6**	**P7**	**P8**
Date	26 July−28 July	28 July −04 Aug	04 Aug−19 Aug	19 Aug−26 Aug	26 Aug−09 Sep	09 Sep−16 Sep	16 Sep−23 Sep	23 Sep−30 Sep
DAP	43–45	45–52	52–67	67–74	74–88	88–95	95–102	102–109

### Data processing algorithms

After raw data was collected in the field, further processing and analysis were performed in the lab. The data processing and analysis program was developed and implemented in MATLAB 2016b (The Math Works Inc., Natic, MA, USA) on a desktop equipped with an intel I7-6700 CPU 3.40 GHz with 16 GB RAM, running on a Windows 10 Enterprise operating system.

**Figure 3:**
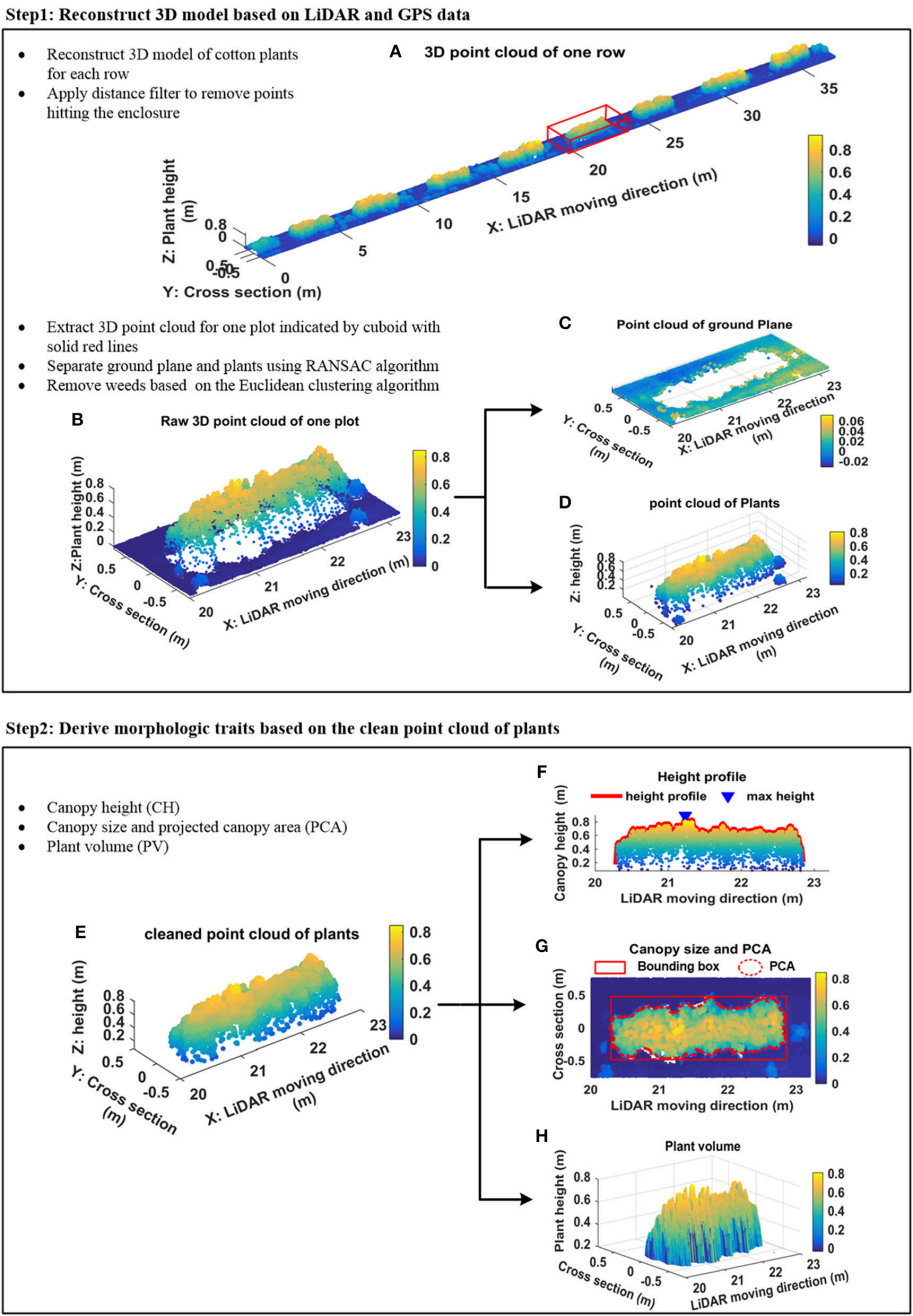
Data processing pipeline. **(A)** Example of 3D point cloud of one scanned row; **(B)** segmented 3D point cloud of a plot indicated by cuboid with solid red lines in **(A)**; **(C)** point cloud of ground plane; **(D)** point cloud of plants; **(E)** denoised point cloud of plants; **(F)** height profile of plants and maximum canopy height; **(G)** projected canopy area; **(H)** plant volume.

Two steps were executed to derive plant features (Figure 3): 3D plant surface model generation (section Generation of 3D Model) and morphologic plant parameter extraction (section Extraction of Morphologic Traits).

#### Generation of 3D model

The 3D model for each row was reconstructed based on GPS and LiDAR data. The GPS and LiDAR dataset was depicted by Equation (1). The two kinds of data were synchronized using timestamps.

(1) $$\begin{array}{c} P_{GPS}=\left\{P_{GPS0},P_{GPS1},\cdots ,P_{GPS\left(N-1\right)}\right\}\\ F_{LiDAR}=\left\{F_{LiDAR0,}F_{LiDAR1,\cdots ,}F_{LiDAR(M-1)}\right\} \end{array}$$

***P***_*GPS*_ was the set of collected GPS data, and ***F***_*LiDAR*_ was the set of scanned frames of LiDAR. The number of GPS points was *N*, and the number of LiDAR frames was *M*.

The distance between two adjacent GPS points, denoted by Δ***P***_*GPS*_, was computed by Equation (2). *f*_*LiDAR*_ and *f*_*GPS*_ were the data acquisition frequency of LiDAR and GPS, respectively. In this study, the data acquisition frequency of GPS was *f*_*GPS*_ = 5 Hz, and LiDAR scanning frequency was *f*_*LiDAR*_ = 50 Hz. Therefore, there were 10 scanned frames, each containing 571 points (the aperture angle was 190° with angular resolution 0.33°) between every two adjacent GPS points (Equation 3). Assuming that the tractor was moving at a constant speed during the interval (200 ms) of two adjacent GPS points, the distance of the two adjacent frames within two adjacent GPS points was computed using Equation (4). Therefore, the position of each LiDAR scanned frame was obtained using Equation (5). ***D***_*offset*_ was the offset between LiDAR and GPS. In this study, ***D***_*offset*_ was fixed during data collection in the field, and the measured point at 0° scanning angle was used to depict the frame position.

(2) $$\begin{array}{c} \{\begin{array}{ll} \Delta {\vec{P}}_{GPS}(i)=P_{GPS}(i)-P_{GPS}(i-1), & i=1,2,...N-1\\ \Delta {\vec{P}}_{GPS}(0)=\vec{0}, & i=0 \end{array} \end{array}$$

(3) $$\begin{array}{c} \alpha =\frac{f_{LiDAR}}{f_{GPS}} \end{array}$$

(4) $$\begin{array}{c} {\vec{d}}_{\text{i}}=\frac{\Delta {\vec{P}}_{GPSi}}{\alpha },i=0,1,2,...N-1 \end{array}$$

(5) $$\begin{array}{c} f_{LiDAR}(k)=f_{LiDAR}(i\alpha +j)=p_{GPS}(i)+j\times {\vec{d}}_{i}+{\vec{D}}_{offset}\\ \;i=0,1,2,...N-1,j=0,1,2...9,k=0,1,...M-1 \end{array}$$

More details related to this processing can be found in Sun et al. (2017). Figure 3A shows an example of the reconstructed 3D model of one scanned row. A distance filter was used to remove points hitting the enclosure. A plot level 3D model was extracted from the 3D model of the row according to the proportions of the field layout (Figure 3B). The standard RANSAC algorithm was applied to cut-off points of the ground plane (Figures 3C,D). An Euclidean clustering algorithm (Rusu et al., 2008) was used to remove points generated by weeds that were not attached to cotton plants (Figure 3E).

#### Extraction of morphologic traits

The maximum canopy height (CH) was measured by calculating the distance from the ground plane to the apex of all measured points (Figure 3F). In addition, different percentiles canopy height—from 85th to maximum CH with steps of 3%—were calculated based on the histogram of the *z* coordinate for all measured points. The boundary points of the plant canopy were detected by projecting all points onto the ground plane, and the projected canopy area (PCA) and bounding box of canopy structure (representing maximum length and width occupied by the canopy) were extracted from boundary points (Figure 3G). A Trapezoidal rule based algorithm was used to calculate plant volume (PV) (Figure 3H) in order to provide an indication of the 3D space occupied by each plot.

**Figure 4:**
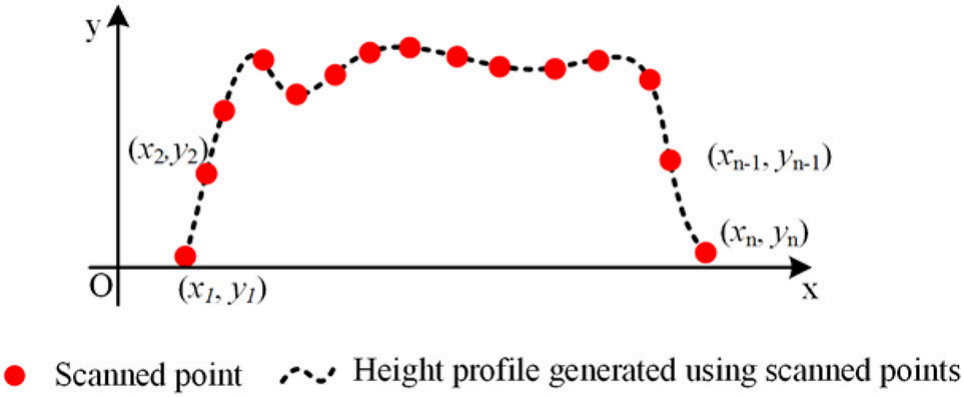
Estimated area (the area under height profile curve) using Trapezoidal rule.

***S*** denoted the line scan set for a plot (Equation 6) which contained *k* line scans. For a scan *s*_*i*_ which contained *n* measured points, the dashed line was the surface profile and the red spots were measured points by LiDAR (Figure 4). The area denoted as *A*_*i*_ under the line scan *s*_*i*_ could be estimated using the measured points (red spots in Figure 4) based on Trapezoidal rule (Equation 7), where (*x*_*i*_, *y*_*i*_) were the coordinates of the *i*th measured point.

(6) $$\begin{array}{c} S=\left\{s_{1},s_{2},\cdots ,s_{k}\right\} \end{array}$$

(7) $$\begin{array}{c} A_{i}=\sum_{j=1}^{n-1}\left(x_{j+1}-x_{j}\right)\left(\frac{y_{j+1}+y_{j}}{2}\right) \end{array}$$

Therefore, the area set denoted as ***A*** for the plot was obtained using Equation (8).

(8) $$\begin{array}{c} A=\left\{A_{1},A_{2},\cdots ,A_{k}\right\} \end{array}$$

Similar to the area calculation process, PV was obtained with Equation (9).

(9) $$\begin{array}{c} \text{PV}=\sum_{i=1}^{k-1}\left(l_{i+1}-l_{i}\right)\left(\frac{A_{i+1}+A_{i}}{2}\right) \end{array}$$

*l*_*i*_ was the position along the tractor moving direction of the *i*th line scan.

### Validation experiments

To verify the accuracy of canopy height measurements using point cloud data, 96 samples of maximum CH—the perpendicular distance from the highest point to the ground plane—were manually measured using a tape measure during data collection in the field. The samples were measured on four different days: July 28, August 04, August 26, and September 09, 2016. On those days, the average wind speeds were around 2.7, 3.4, 2.3, and 2.5 m/s, respectively.

For PCA validation experiments, eight “dummy plants” were made with different canopy shapes using printed paper leaves and metal wires. The dummy leaves were in three different sizes–10, 41, and 92 cm^2^–and there were six of each size. The leaves of two dummy plants were arranged to be overlapped. Each dummy plant was scanned by LiDAR and imaged by a DSLR camera (FUJIFILM X-A10 mirrorless camera, FUJIFILM, Tokyo, Japan). A scale bar was used to calibrate the real size of the plant. The ground truth of PCA was computed by segmenting leaves based on color information.

For PV validation experiments, eight plants at different growth stages were used, among which five were real cotton plants, one was a shrub plant, and two were dummy plants. The six real plants were in the leaf and canopy development growth stage, whereas the two dummy plants were used to mimic flowering and boll development growth stages. Each plant canopy was divided into 50 mm cylindrical discs from the top to the bottom and the diameter of each disc was manually measured using a tape measure. The diameter and height of each cylindrical disc were used to estimate the volume of each disc, and then the volume of the whole plant was obtained by summing the volumes of each disc. Manual measures of the three traits were plotted vs. point cloud estimates, and regression analysis was used to compute the root mean square error (RMSE) and coefficient of determination (*R*^2^). More information about PCA and PV validation experiments can be found in supplementary materials.

### Plant growth analysis

Plant growth dynamics were obtained by monitoring plants over the growing season when plant morphologic traits were extracted. Growth trends and growth rates for each morphologic trait noted above (CH, PCA, PV) were computed and analyzed over the monitoring period. Growth trends were determined as the variation in measured traits over the monitoring period. The results were given as mean values and standard deviations of all morphologic parameters. A three-parameter logistic model (3PLM) was used to fit growth curves of the three detected traits for each cultivar (Tessmer et al., 2013). The model is a function of time *t* as shown in Equation (10).

(10) $$\begin{array}{c} y\left(t\right)=\frac{x_{0}x_{n}}{x_{0}+(x_{n}-x_{0})e^{-\tau (t-T)}} \end{array}$$

where *t* was the time which was denoted by days after planting in this study, τ was a coefficient, *y*(*t*) was detected traits at time *t, x*_0_, *x*_*n*_ were the initial value and the upper horizontal asymptote of the detected trait, respectively, and *T* was the first day of data collection which was also denoted by days after planting (in this study *T* = 45). *x*_0_, *x*_*n*_, and τ can be estimated using non-linear least squares based on the observations of the detected traits.

Growth rate was determined as the average change in measured traits over a time interval. Growth rate was calculated by Equation (11).

(11) $$\begin{array}{c} GR=\frac{P_{t}-P_{t-\Delta t}}{\Delta t} \end{array}$$

where *GR* was the growth rate, Δ*t* was the time internal, and *P*_*t*_ was the measured plant trait at time *t*.

### Correlation analysis between morphologic traits and yield

Seed cotton (mature fiber plus seeds to which it was attached) was harvested manually on November 4, 2016, and yield was expressed as g/plot. In order to explore the relationship between derived morphologic traits and final yields, linear regression analysis was conducted for each cultivar over the monitoring period. The coefficient of determination (*R*^2^) was computed.

## Results

### Morphologic traits extraction and validation

**Figure 5:**
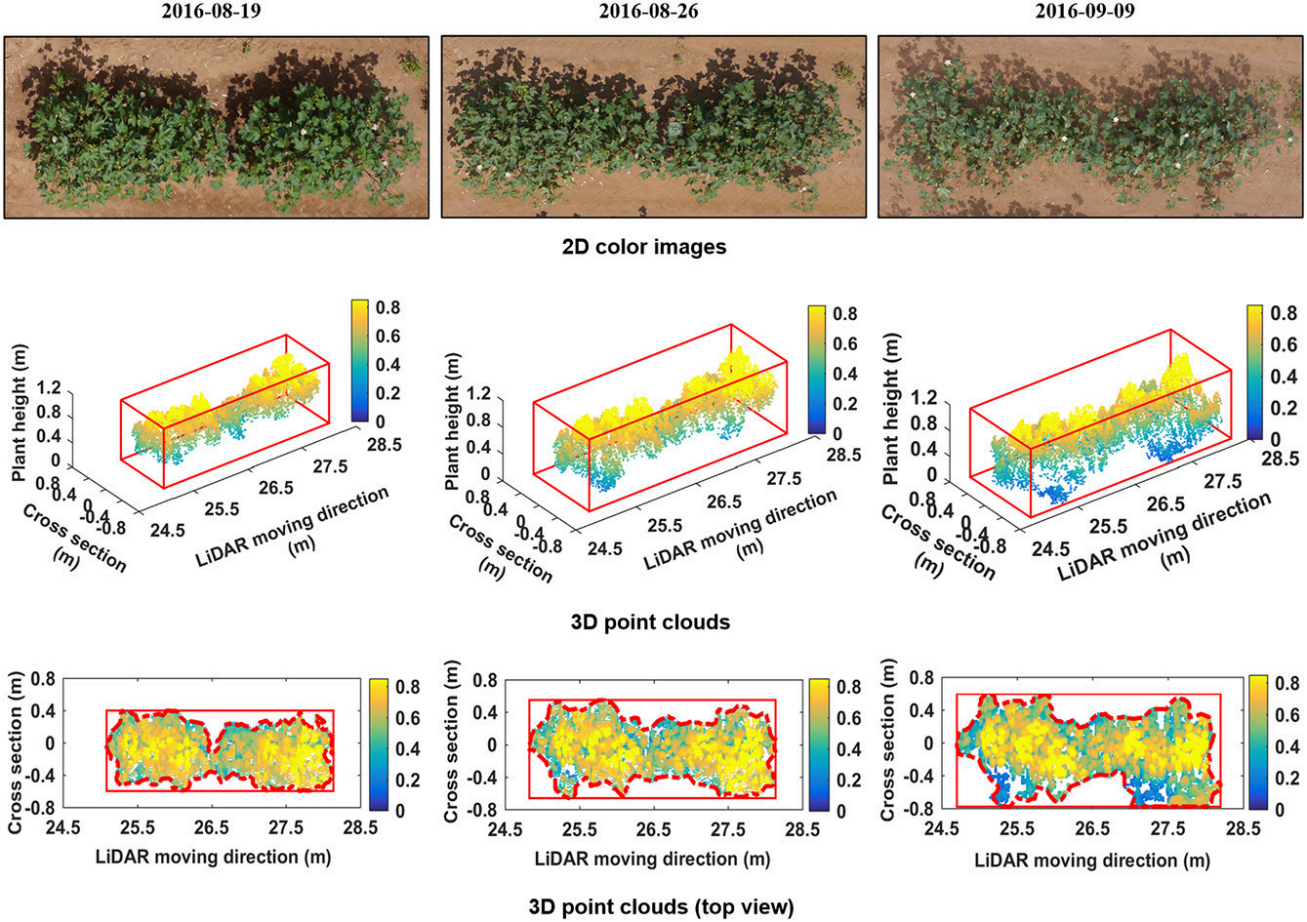
Reconstructed 3D point cloud of one plot (Plot ID: row 8, column 6) and its evolution from August 19 to September 09, 2016. The first row shows 2D color images taken from above. The second row shows the reconstructed 3D point clouds of cotton plants. The third row shows height maps of cotton plants, obtained by projecting all points on the ground plane (color indicates height).

Morphologic trait information of cotton plants was derived from the reconstructed 3D point clouds. Figure 5 shows the 3D point clouds over time compared to the 2D color images of the same plot. The reconstructed 3D point clouds on August 19, August 26, and September 9, respectively, were obtained using 207, 256, and 263 scans and contained 11,403, 14,379, and 14,485 points. The cuboids indicated by solid red lines for graphs in row 2 of Figure 5 are 3D bounding boxes and points within the cuboids belong to plants. The rectangles indicated by red solid lines for graphs in row 3 of Figure 5 are 2D bounding boxes, and the dashed red lines are the detected boundaries from which PCA was derived. Morphologic traits including CH, PCA, and PV were extracted from the 3D point clouds.

**Figure 6:**
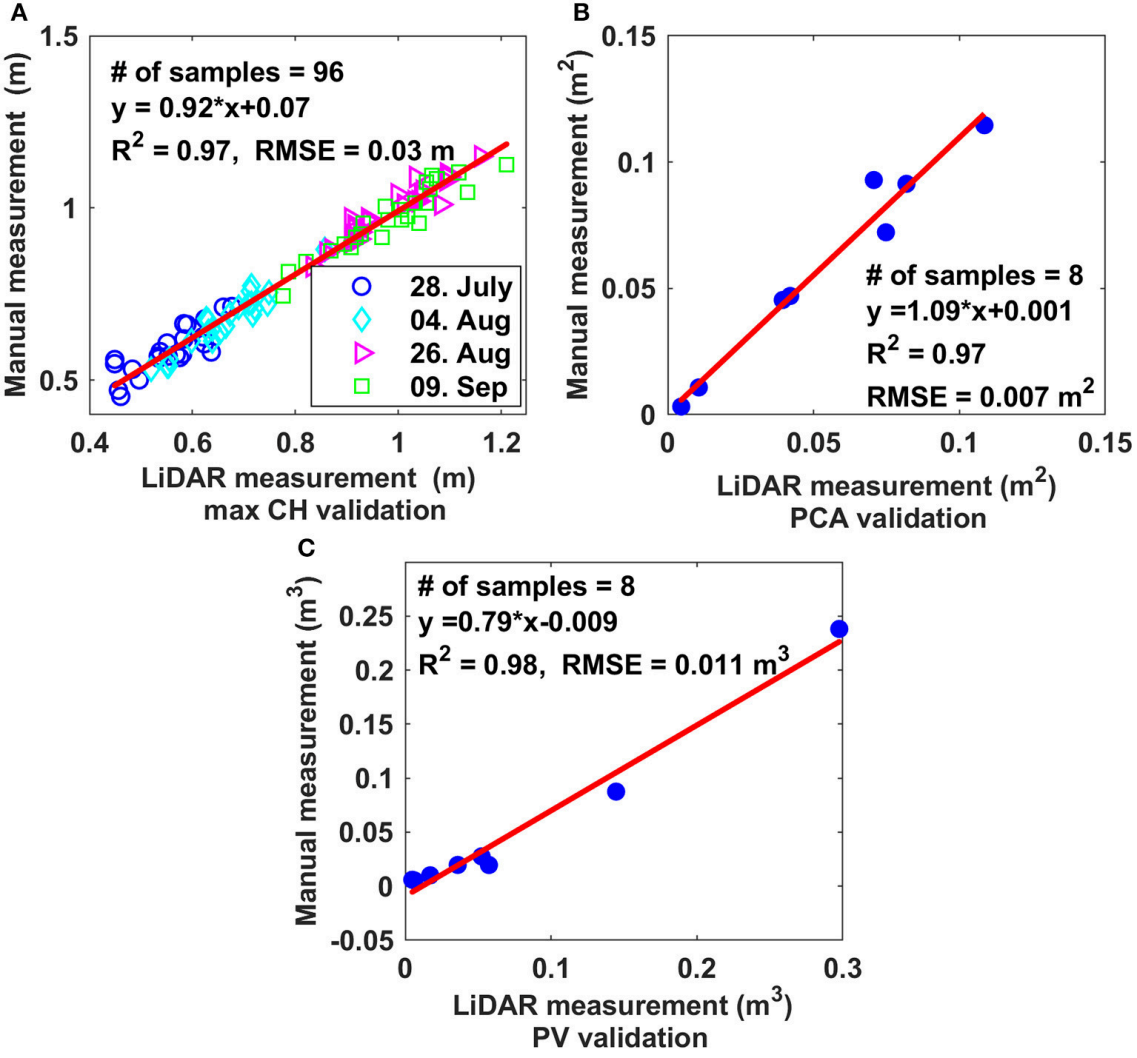
Comparison of three derived morphologic traits based on LiDAR data with ground-truthing. **(A)** Maximum canopy height; **(B)** projected canopy area; **(C)** plant volume.

CH-, PCA-, and PV-values derived from LiDAR data were highly correlated with manually measured values, with *R*^2^-values of 0.97, 0.97, and 0.98 and RMSE values of 0.03 m, 0.007 m^2^, and 0.011 m^3^, respectively (Figure 6).

**Figure 7:**
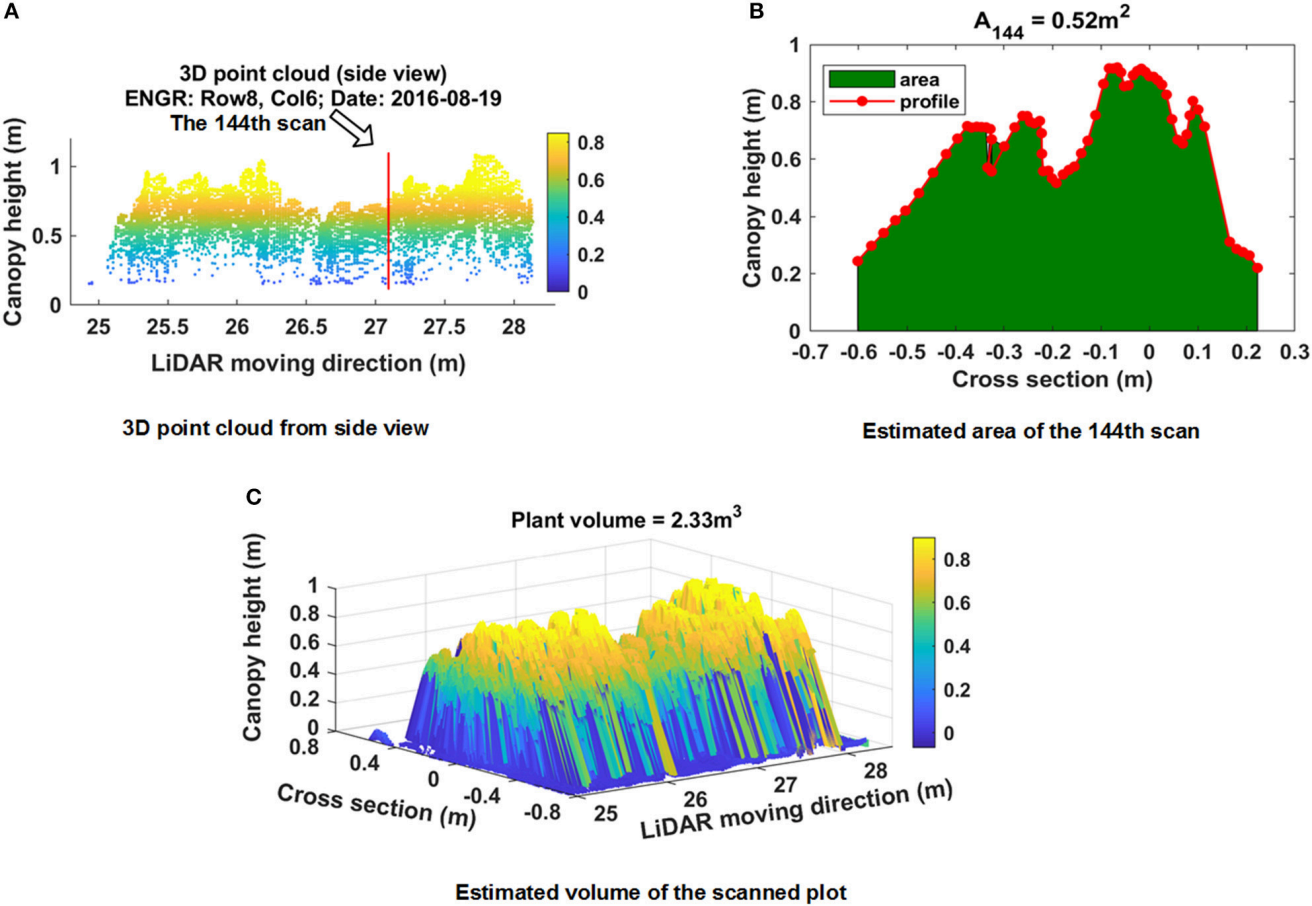
Example of plant volume computation of a plot (plot ID: row 8, column 6, Date: August 19, 2016) using the proposed Trapezoidal rule based method. **(A)** Side view of 3D point cloud; **(B)** estimated area under the profile of scan 144; **(C)** computed plant volume.

Figure 7A shows the side view of the point cloud of the plot presented in Figure 5. The data was collected on August 19 and consisted of 207 line scans. The number of measured points for each line scan varied depending on the width of plants. A total of 66 points were measured for the 144th scan (Figure 7B). Points near the center were denser than points located at two terminal areas because the inter-distance between two adjacent points increased with increasing distance between the LiDAR and measured points. A minimum threshold of 0.05 m was applied to remove points. Based on the proposed PV calculation method, the area under the profile and PV were estimated: A_144_ = 0.52 m^2^, PV = 2.33 m^3^ (Figure 7C).

### Plant growth analysis

**Figure 8:**
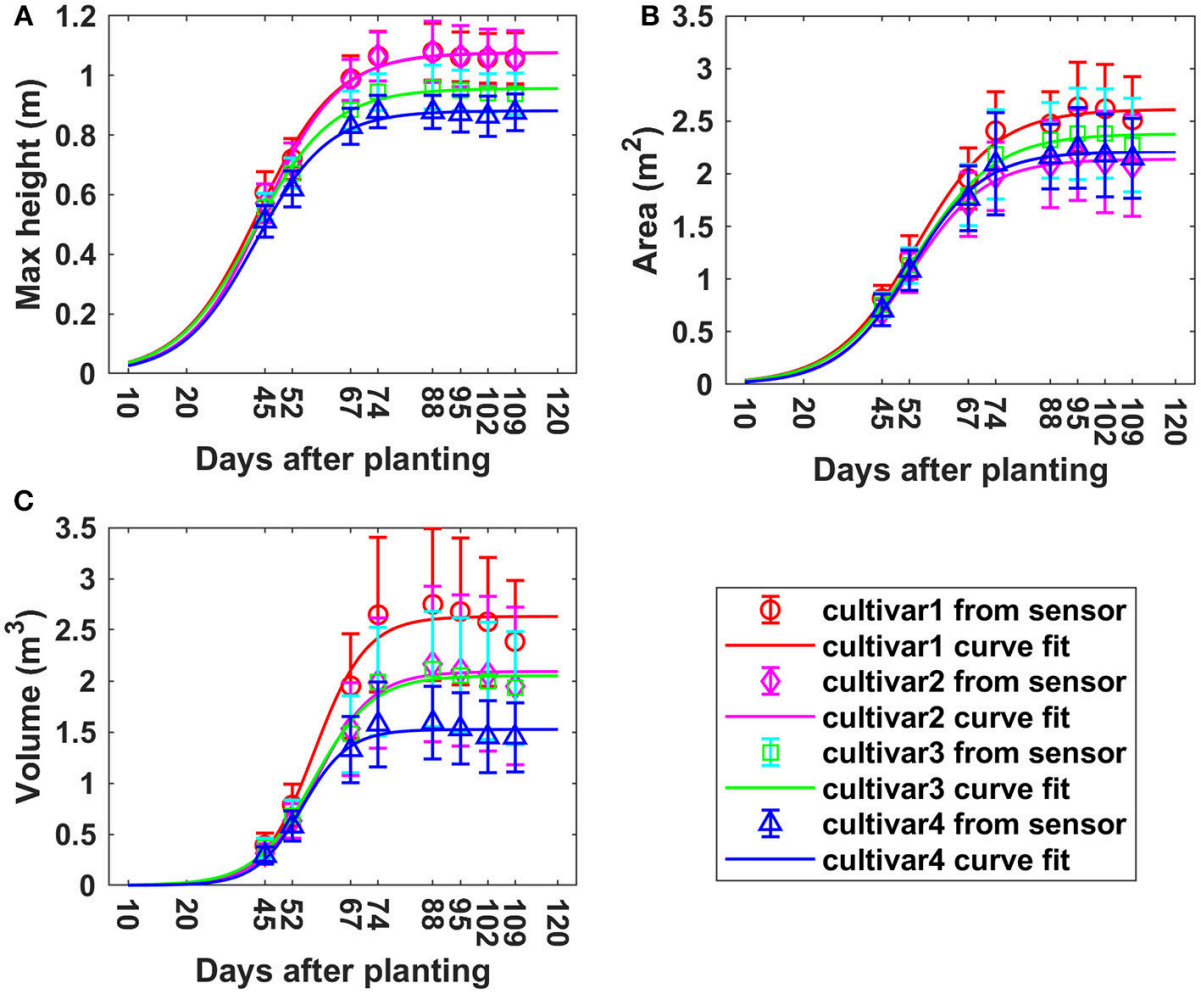
Growth curves for derived morphologic parameters over the monitoring period. **(A)** Maximum canopy height; **(B)** projected canopy area; **(C)** plant volume.

Overall, the three measured morphologic traits—CH, PCA and PV—showed similar growth trends over the monitoring period based on the measurements from the sensor, but the actual value was cultivar specific for each trait (Figure 8). For maximum CH, all cultivars reached the maximum height on around day 88. Cultivar 1 and 2 had similar average maximum heights of 1.08 m, while cultivars 3 and 4 reached the peak values of 0.96 and 0.88 m, respectively. Cultivar 1 and 2 were around 22.7% higher than cultivar 4. PCA continued to increase until around 95 DAP, which was 7 days longer comparted to maximum CH. The maximum PCAs of cultivars 1, 2, 3, and 4 were 2.73, 2.23, 2.47, and 2.34 m^2^, respectively. Cultivar 1 had much larger PCA-value than the other three cultivars which had similar PCA. It is around 22.4% larger than cultivar 2 which had the minimum area. Cultivar 2 showed the highest CH, but lowest PCA over the whole monitoring period due to less horizontal canopy expansion in the horizontal direction. For the growth curve of PV, the maximum points for all four cultivars were reached on day 88, they were 2.75, 2.17, 2.11, and 1.59 m^3^ for cultivar 1, 2 3, and 4. Larger differences among cultivars were observed for PV than PCA. The maximum volume was around 73.0% larger than the minimum one. In summary, cultivar 1 had the largest canopy (highest PV, CH, and PCA) while cultivar 4 had the lowest maximum CH and PV. Although cultivar 2 had high maximum CH, its PCA was the lowest, making its PV in the middle range among the cultivars. The derived values and variation of morphologic traits over time were in good agreement with the developmental phases of cotton (Ritchie et al., 2007).

Growth curves generated for each cultivar of all three detected traits showed good correlation with the sensor measurements. The date when the traits reached their upper horizontal asymptotes and the values of their upper horizontal asymptotes were similar to the results obtained from measured data. The growth curve varied little after reaching the upper horizontal asymptotes, while the sensor measurements showed a decreasing trend which was mainly due to defoliation. Supplementary Table 1 presented estimated parameters of the 3 PLM and their 95% confidence interval (CI).

**Figure 9:**
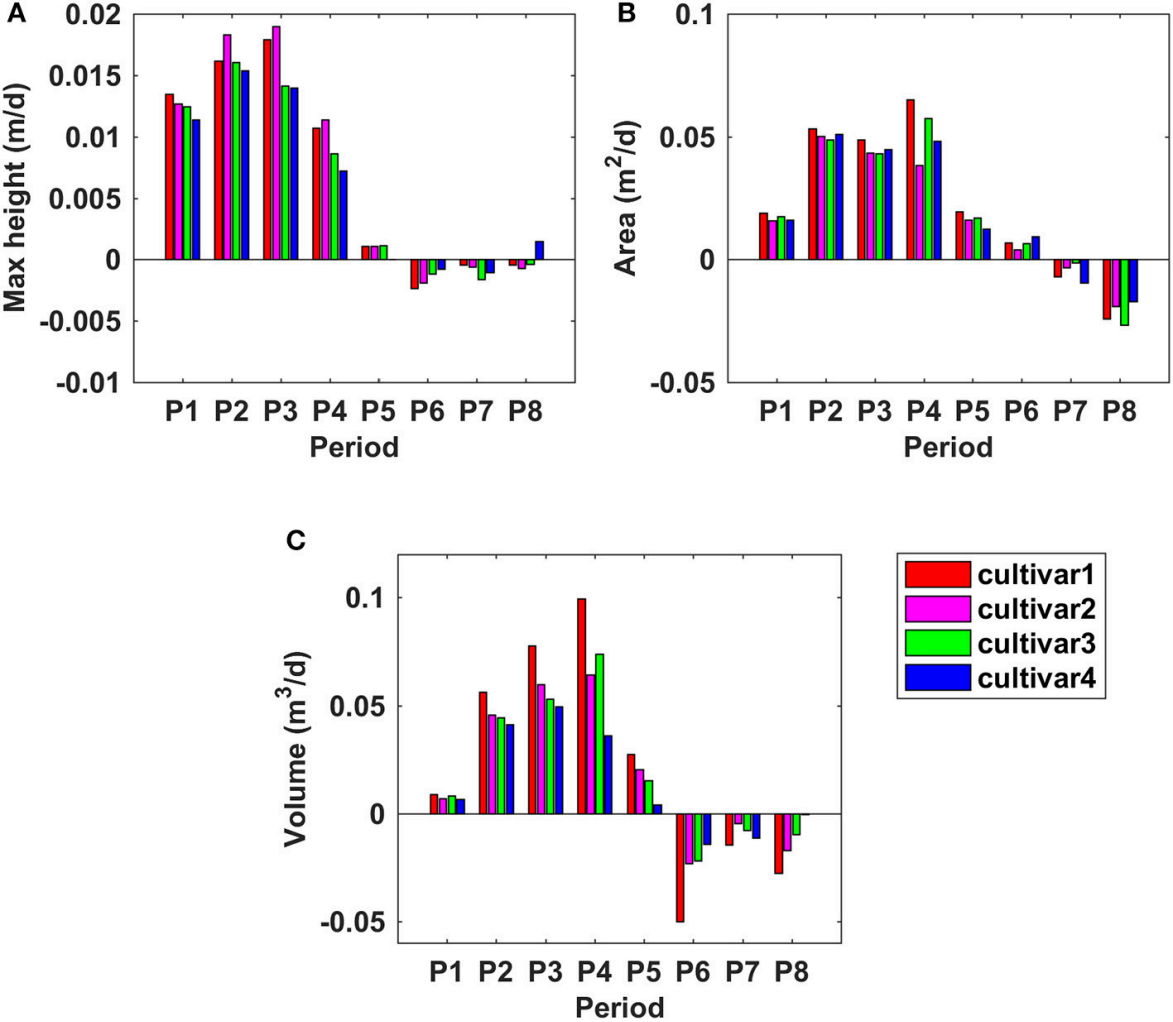
Growth rates for derived morphologic parameters at different time frames during the monitoring period. **(A)** Maximum canopy height; **(B)** projected canopy area; **(C)** plant volume.

Figure 9 shows the growth rates of maximum CH, PCA, and PV at different periods of time. For maximum CH, cultivars 1 and 2 had similar growth rates, and grew faster than cultivars 3 and 4 during the period from P1 to P5. The peak of GR of cultivar 1 and 2 was observed during P3 (between 52 and 67 DAP), which was around 0.018 m per day (m/d); However, it was during P2 for cultivar 3 and 4, which was around 0.015 m/d (Figure 9A). For PCA, all four cultivars had similar GR during P2 and P3; However, large differences emerged during the period from P3 to P6. The peak of GR for cultivar 1 and 3 was in P4, with the values of 0.065 m^2^/d; while it was in P2 for cultivar 2 and 4, with the same value of 0.054 m^2^/d. PCA increased until P6 (95 DAP) and then started to decrease (Figure 9B), 21 days later than the period when the maximum CH started to decrease. During the PCA increasing period, cultivar 1 grew faster than the other three. PV had a faster GR than the other three cultivars during P1 to P4—it reached the peak in P4 with the value of around 0.1 m^3^/d. Cultivar 2, 3, and 4 reached the peak of GR in P4 (0.06 m^3^/d), P4 (0.07 m^3^/d), and P3 (0.05 m^3^/d), respectively. In summary, the fastest GRs for all three traits were during P2 to P4, that was from 45 to 74 DAP, which was also indicated by the growth curves in Figure 8.

ANOVA-tests showed that cultivars had a significant influence on the derived morphologic traits over the monitoring period (Table 2). This allowed us to utilize regression analysis to determine which morphologic traits and measurement times were most closely associated with final yield for each cultivar.

**Table 2:** Effects of cultivars on derived parameters over the monitoring period (Year: 2016).

	**July 28 (DAP 45)**	**Aug 04 (DAP 52)**	**Aug 19 (DAP 67)**	**Aug 26 (DAP7 4)**	**Sep 09 (DAP 88)**	**Sep 16 (DAP 95)**	**Sep 23 (DAP 102)**	**Sep 30 (DAP 109)**
Max CH	<0.001^**^	<0.001^**^	<0.001^**^	<0.001^**^	<0.001^**^	<0.001^**^	<0.001^**^	<0.001^**^
PCA	0.003^**^	0.003^**^	0.034^*^	0.005^**^	0.001^**^	0.001^**^	0.002^**^	0.006^**^
PV	0.002^**^	0.001^**^	<0.001^**^	<0.001^**^	<0.001^**^	<0.001^**^	<0.001^**^	<0.001^**^

* *Significant at the 0.05 probability level*,

** *Significant at the 0.01 probability level*.

### Relationship between morphologic traits and yield

A significant difference in the final yield was observed between the four cultivars (Table 3). Cultivar 2 produced significantly lower yields than cultivar 3 and 4.

**Table 3:** Differences in final yield between four cultivars.

**Cultivar**	**Mean yield (g/plot)**
Cultivar1	928.51^AB^
Cultivar2	781.58^B^
Cultivar3	954.18^A^
Cultivar4	937.22^A^

*p < 0.05. Different letters indicate significant differences between cultivars*.

**Figure 10:**
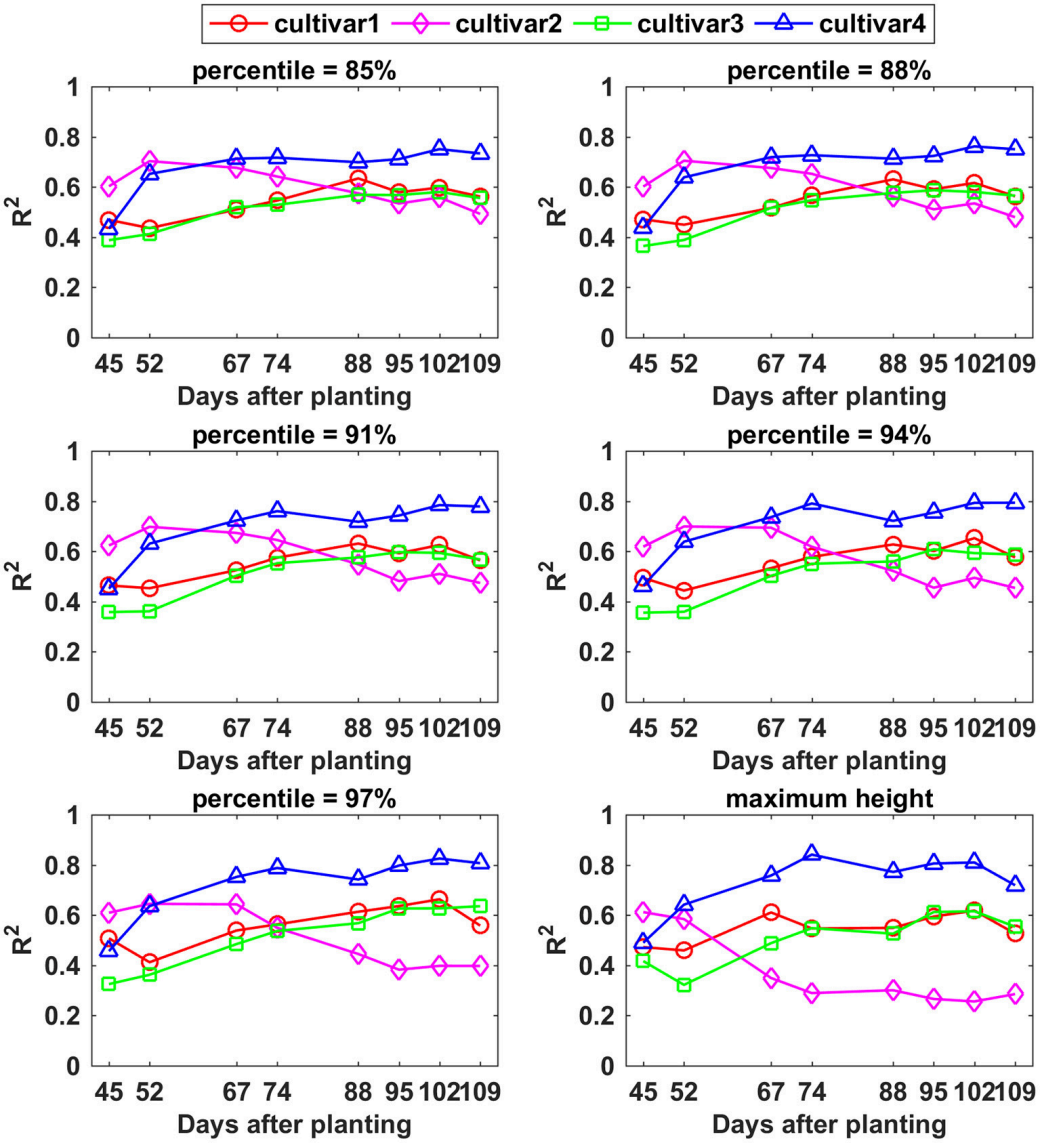
Correlation analysis results between different percentiles of canopy height and yield by days after planting for each cultivar.

The relationship between CH and final yield showed similar trends using data from various CH percentiles, especially between the 85th and 94th percentiles. The *R*^2^-values for cultivar 1, 3, and 4 increased over the monitoring period although there existed only slight variation, whereas cultivar 2 exhibited a decreasing trend (Figure 10). Among the cultivars studied, cultivar 4 had the highest *R*^2^-values (up to 0.84) from day 67 on, based on the 85th CH to maximum CH. The variation curve of cultivar 1 was analogous to the curve for cultivar 3, both reaching the highest *R*^2^-values between 88 and 95 DAP. In contrast, cultivar 2 demonstrated the highest correlation with yield at an early growth stage around 52 DAP. Based on the results from this study, percentiles from 85 to 94% of CH during the period from 67 to 109 DAP are recommended for yield estimation application due to not only high but also stable *R*^2^-values for all four cultivars.

**Figure 11:**
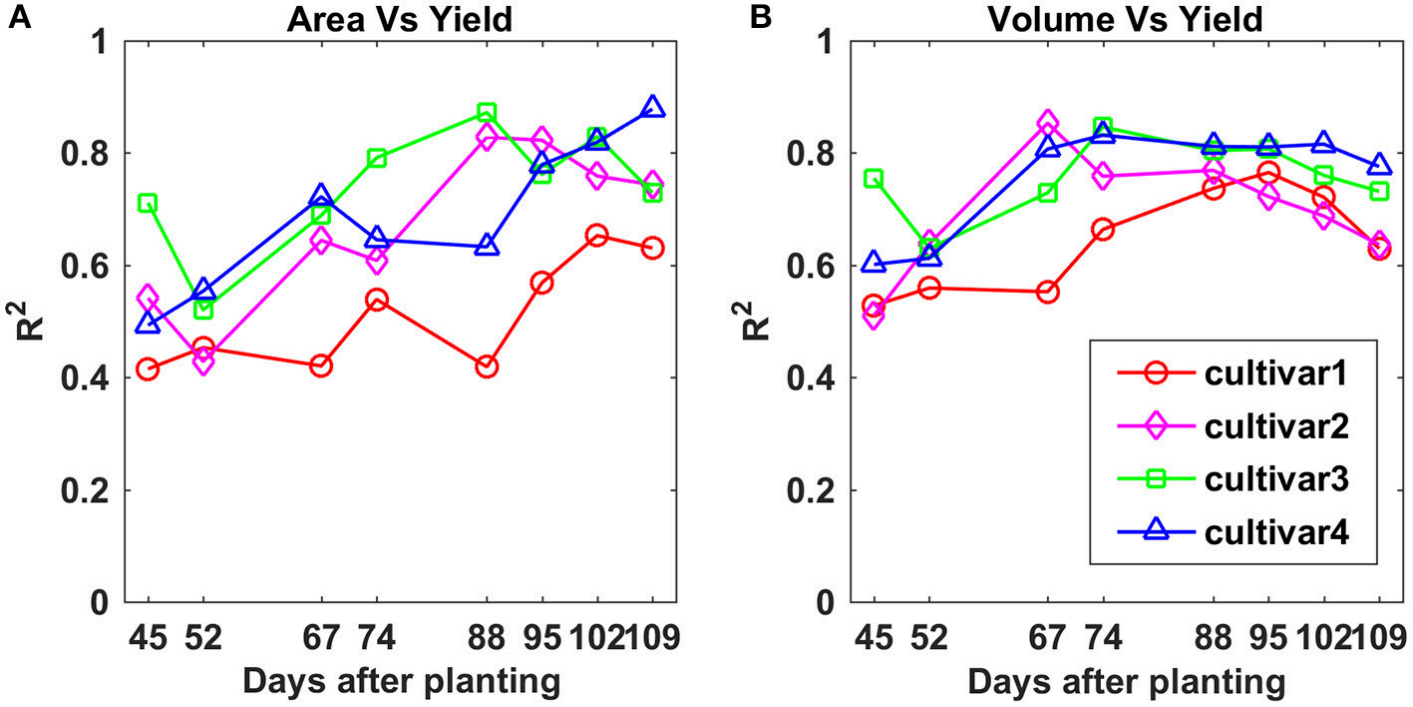
Correlation analysis results between **(A)** projected canopy area and yield, and **(B)** plant volume and yield by days after planting for each cultivar.

Overall, the *R*^2^-values between PCA and final yield increased over the monitoring period for all four cultivars (Figure 11A). The *R*^2^-values for cultivars 2, 3, and 4 on each data collection day were similar, while cultivar 1 exhibited lower *R*^2^-values. Compared to CH parameters, a major difference was that cultivar 2 had an opposite variation trend. The highest *R*^2^-values for all four cultivars were reached between 88 and 109 DAP, and they were 0.65, 0.83, 0.87, and 0.88 for cultivars 1, 2, 3, and 4, respectively. This indicated that PCA was more closely related to final yield than CH. For both CH and PCA, cultivar 4 showed the strongest correlation with final yield among the four cultivars.

For PV, the difference in *R*^2^-values between the four cultivars became smaller over the monitoring period compared to CH and PCA, especially between 88 and 102 DAP (Figure 11B). The maximum *R*^2^-values were 0.77, 0.85, 0.84, and 0.83 on 95, 67, 74, and 74 DAP for cultivars 1, 2, 3, and 4, respectively, which were similar to the values for PCA, and better than CH. This indicated that PV was a more stable trait than CH and PCA and could feasibly be used to predict cotton final yield.

**Figure 12:**
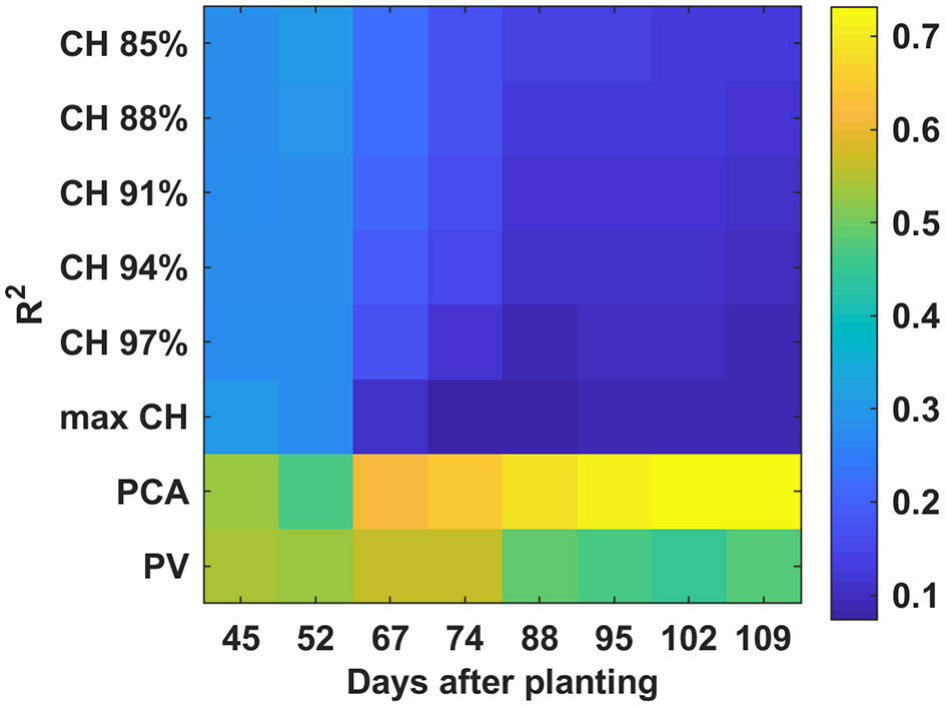
Correlation analysis results between derived parameters and yield with all cultivars combined by days after planting.

PCA and PV had higher *R*^2^-values than CH over the monitoring period (Figure 12) when combining all cultivars. The *R*^2^-values increased steadily to a final value of 0.72 for PCA. PV reached the highest *R*^2^-value (0.56) on 67 DAP—although the PV *R*^2^-values were less than those of PCA from 67 to 109 DAP, they were more stable than those of PCA. The lowest correlation was found between CH and final yield. A decreasing trend was observed from 45 to 109 DAP which was mainly due to cultivar 2.

## Discussion

We have demonstrated that 3D point clouds reconstructed based on data collected by a 2D LiDAR and an RTK-GPS, and associated data processing methodology, accurately estimated specific morphologic traits from cotton plants under field conditions. The precise 3D point cloud, which was the basic dataset for analysis, was critical for the successful extraction of plant morphologic traits (Paproki et al., 2012; Paulus et al., 2014b; Duan et al., 2016). The reconstructed 3D model visually represented plant canopy structures (Figure 5). The CH, PCA, and PV derived by our system were highly correlated (the slopes were close to one) with those measured manually (Figure 6).

The system demonstrated great potential for high-throughput phenotypic analysis. The number of points for each plot varied from 2,000 to 20,000 over the monitoring period, depending on the plant size. The tractor was driven in the field at a speed of around 0.5 m/s for data collection, taking about 6 s to scan one plot that contained 15 plants. Thus, one could anticipate scanning about 600 such plots per hour. The average time consumed for 3D reconstruction and parameter extraction was around 5.27 s per plot so about an hour for the aforementioned 600-plot field. Therefore, both data acquisition and data analysis speeds are suitable for application to field experiments of the sizes used in many breeding programs. The system also has great potential to implement online data analysis with faster computing power in the future, which is particularly useful for large field applications.

The high throughput phenotypic analysis by a LiDAR-based method has several advantages compared to image-based methods. Paproki et al. (2012) conducted cotton plant morphologic trait analysis using an image-based 3D reconstruction method, requiring about 7 min to collect images for each plant and an average processing time of 15 min for 3D reconstruction work of each plant. Additionally, image quality could be significantly affected by highly variable illumination conditions (Nuske et al., 2014), which limited its in-field applications. In contrast, LiDAR is more versatile in a wide range of light conditions since it is equipped with its own light source. For manual operation, ~30 min were required for a typical analysis per plant depending on the size and complexity, and destructive harvests were often required (Paproki et al., 2012).

The high throughput of the proposed system and its non-invasive features permit data acquisition repeatedly over the growing season, opening the door to acquisition of plant growth rate variation, which is valuable for a wide range of applications such as building plant growth models (Tessmer et al., 2013; Weraduwage et al., 2015), exploring factors influencing growth processes (Rahaman et al., 2015; Awlia et al., 2016), genomics-assisted crop breeding (Watanabe et al., 2017), and QTL analysis (Bac-Molenaar et al., 2015; Cui et al., 2017). In this study, all plants in the experimental field were scanned from 43 to 109 DAP. Plant growth curves were generated and correlation between derived parameters and the final yield were explored, which could be used for yield prediction (Sharma et al., 2015) and species identification (Remagnino et al., 2016). Thus, the system described herein not only provides an indication of which LiDAR-derived parameters are most closely associated with yield, but also allows us to define the period during the growing season in which a morphologic trait has the greatest correlation with final yield.

While CH in the current study was highly correlated with within-cultivar yield variation in some instances (i.e., cultivar 4 on later sample dates), it should be noted that correlations between CH and yield for all cultivars combined were substantially lower than those obtained for other derived parameters such as PCA and PV. This is not surprising since the cotton crop exhibits a quadratic response of yield to CH (Sui et al., 2012), where internode elongation must often be controlled using exogenously applied plant growth regulators (PGRs) in real-world production scenarios (Dodds et al., 2010). While CH may not be inherently predictive of genotypic differences in yield, the deployment of high-throughput methods for estimating maximum CH and rate of change in CH could strongly influence cultivar-specific PGR management strategies. Specifically, cultivars with rapid plant height development typically require more aggressive management strategies (Collins, 2013).

PCA and PV were more strongly associated with yield than CH when considered across all cultivars, suggesting these parameters might have greater utility as high-throughput phenotyping tools to identify potential differences in productivity. The impact of canopy development on yield is easily understood when yield is expressed as the product of total intercepted photosynthetically active radiation, radiation use efficiency, and harvest index (Monteith and Moss, 1977; Monteith, 1994; Earl and Davis, 2003; Stöckle and Kemanian, 2009). Within this conceptual framework, the amount of intercepted photosynthetically active radiation during a growing season will be strongly impacted by the length of the growing season and the leaf area available to intercept solar radiation. Thus, PCA may serve as a suitable proxy for leaf area magnitude and persistence throughout the growing season. PV incorporates both CH and PCA, which could potentially be related to both the amount of leaf area available to intercept incoming solar radiation and the three dimensional space available for developing fruit. Both of these considerations are important given the indeterminate nature of cotton. As fruit development progresses, vegetative growth slows, and Constable and Bange (2015) have clearly illustrated that the ability of the cotton crop to attain high yield potential will be influenced by total leaf area and number of fruiting sites available prior to the development of a large fruiting load on the plant. Thus, it is not surprising that PCA and PV were correlated with yield for much of the growing season. Future work should couple the aforementioned measures with high-throughput assessment of fruit development as fruiting dynamics can drastically impact final yield in cotton (Constable and Bange, 2015).

The top view scan setting of LiDAR provided precise 3D surface models of plants; however, organs under the canopy could not be reached due to plant occlusion. This does not reduce the accuracy of CH and PCA measurements, but hinders the estimation of PV by our system, especially when plant structure becomes complex. Multi-view scan is a commonly used method to reduce plant occlusion effects (Paulus et al., 2014b), and in our future studies two side view scans could be added. In addition, algorithms such as plant shape models (Pastrana and Rath, 2013) have been proposed to overcome plant occlusion effects. However, when plant structure complexity increases, especially at the mature stage with a greatly increased number and size of leaves and stems, occlusion effects would still be present. Plant occlusion remains a challenge for plant phenotyping, especially under field conditions with limited inter-row spacing (Paproki et al., 2012; Paulus et al., 2014a). Wind is another factor which could affect the accuracy of the derived traits since it might result in blurred point clouds. Therefore, calm weather conditions are best for data collection. Based on the CH validation results, our system has a certain robustness against wind influence; wind speed under 3 m/s was feasible. Another limitation was that the tractor speed was restricted by the scanning frequency of the LiDAR (Sun et al., 2017), limiting throughput. While the present system is already compatible with iterative study of thousands of plots, 3D LiDAR technology may greatly reduce this limitation (Weiss and Biber, 2011).

## Conclusion

Precise 3D surface models were reconstructed by the high-throughput phenotyping system developed in this study under field conditions. Multiple morphologic traits at the plot level including plant height, projected canopy area, and plant volume were extracted simultaneously. The system could be used to scan the field repeatedly due to its relatively high data collection and processing capability, which was particularly useful for large field applications. The measured morphologic traits were most highly correlated with final yield during the period between around 67 and 109 DAP. Projected canopy area and plant volume were more closely correlated than plant height to final yield. Future work will focus on involving other sensor data to extract more phenotypic traits from the 3D point cloud. Although this system was solely tested on cotton plants, it is expected to be applicable for use with other crops such as wheat, rice, and soybeans.

## Author contributions

SS, CL, and AP: conceived the idea and designed the experiments; AP and PC: contributed to the preparation of materials and equipment; SS, YJ, RX, and JR: conducted the experiments; SS, YJ, and RX: analyzed the data; SS, CL, AP, JS, and PC: interpreted results and wrote the paper; All authors read and approved the final manuscript.

### Conflict of interest statement

The authors declare that the research was conducted in the absence of any commercial or financial relationships that could be construed as a potential conflict of interest.

## Abbreviations

CH: canopy height
PCA: projected canopy area
PV: plant volume
3PLM: three-parameter logistic model
CI: confidence interval.
