# Supplementary material for: In-field High Throughput Phenotyping and Cotton Plant Growth Analysis Using LiDAR
Source: Front Plant Sci. 2018 Jan 22;9:16. doi: 10.3389/fpls.2018.00016 (PMC5786533; doi:10.3389/fpls.2018.00016)
Supplement: Supplementary file 1 [file Presentation1.PDF]

Supplementary Material

In-field high throughput phenotyping and cotton plant growth analysis using LiDAR

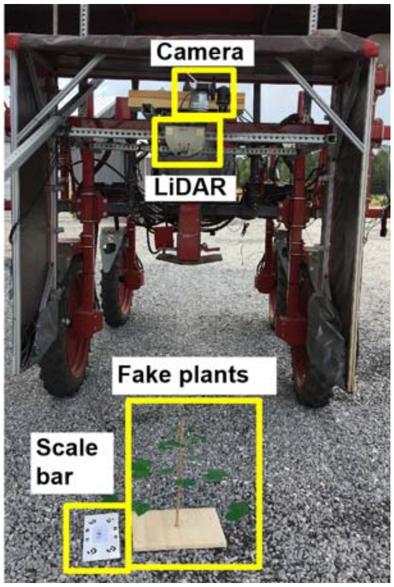

Supplementary Figure 1. PCA validation experiments set-up

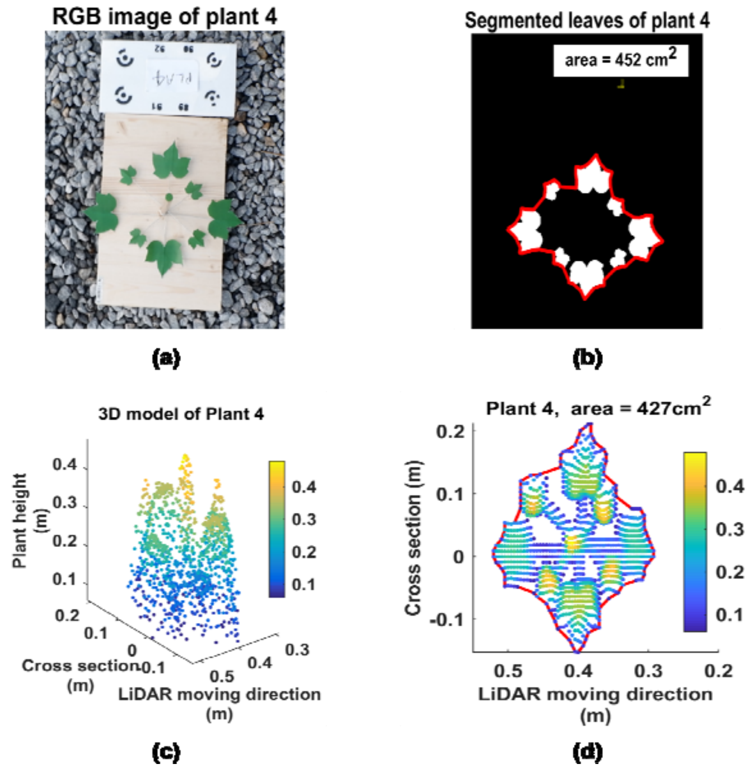

Supplementary Figure 2. Example of comparison of PCA between RGB image based method and LiDAR based method. (a) RGB image of plant 4. The leaves were put on the ground plane; (b) estimated PCA based on the RGB image; (c) 3D model reconstructed using LiDAR data; (d) estimated PCA using LiDAR data. The PCAs in (b) and (d) were the areas within solid red lines.

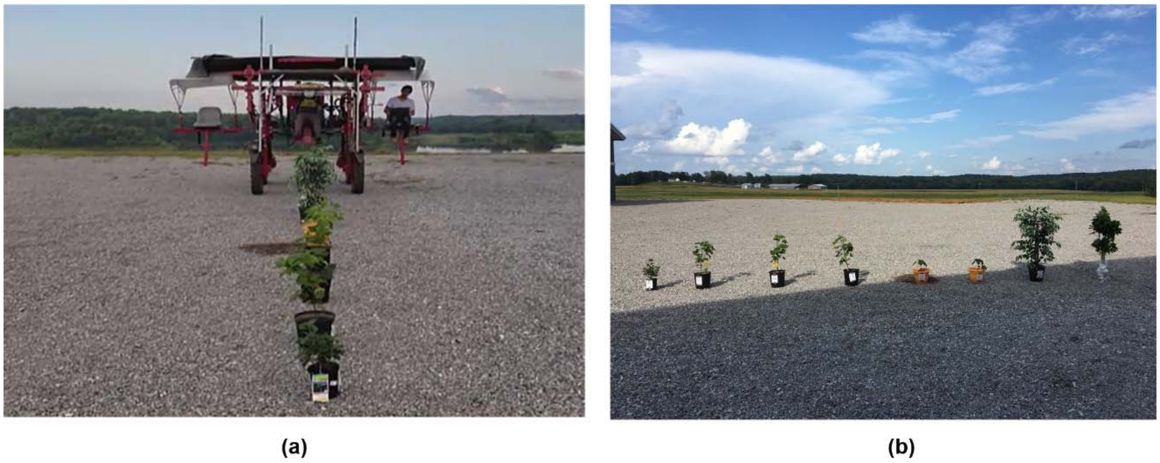

Supplementary Figure 3. PV validation experiments set-up. (a) Platform used for scanning plants; (b) plants used for PV validation experiments

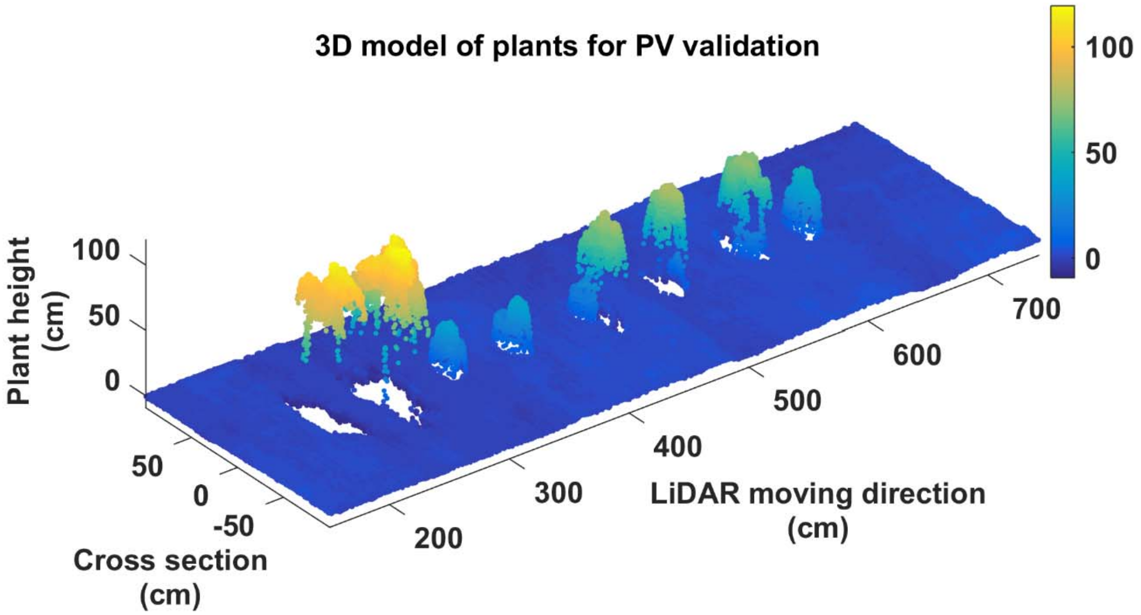

Supplementary Figure 4. 3D model reconstructed based on LiDAR data

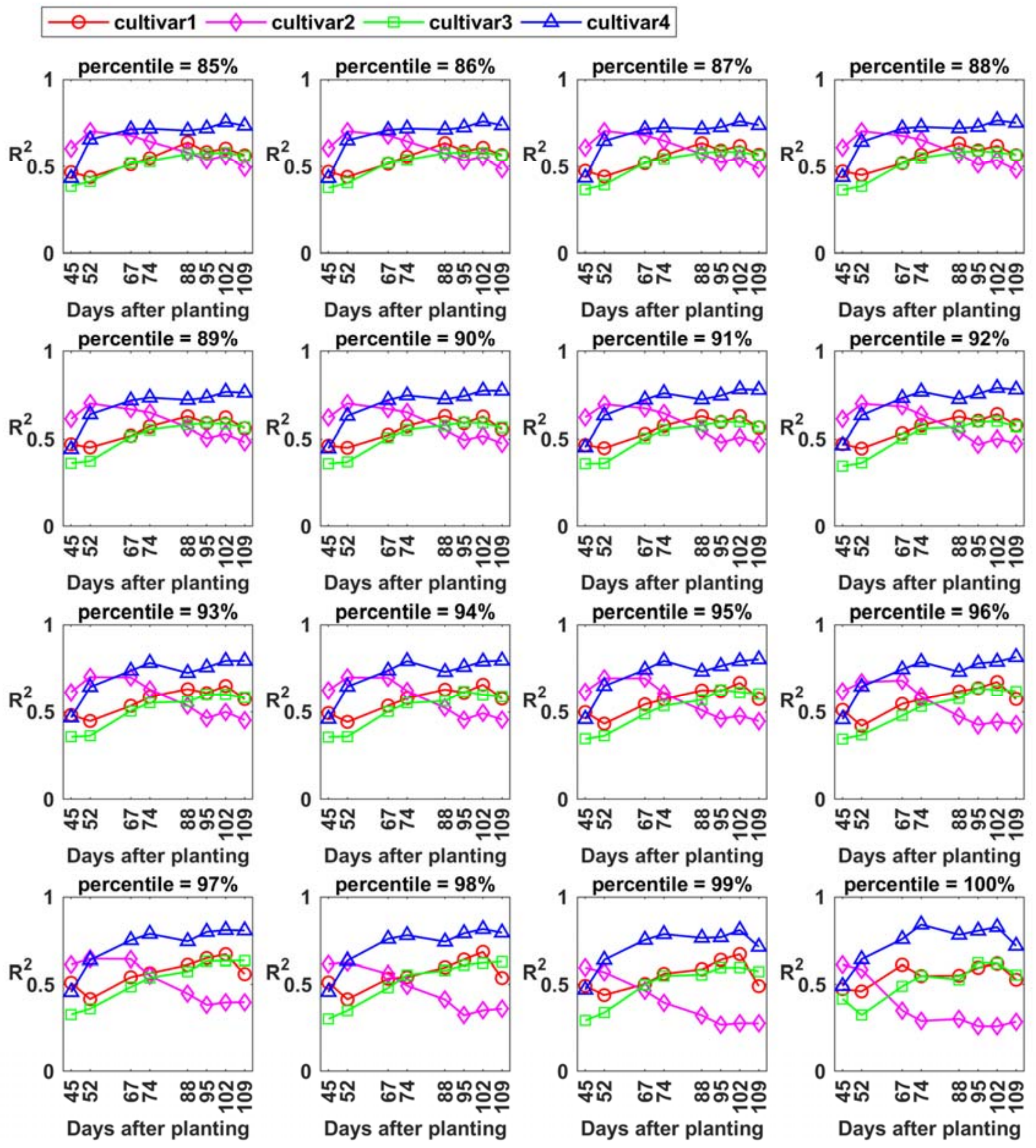

Supplementary Figure 5. Correlation analysis results between different percentiles of canopy height (with a step of 1%) and yield by days after planting for each cultivar.

Supplementary Table 1. Parameters of 3PLM and the lower and upper bounds with 95% confidence

Traits	Cultivar	Parameters			Lower bound of 95% CI			Upper bound of 95% CI		
		x_0	x_n	τ	x_0	x_n	τ	x_0	x_n	τ
MCH	cultivar1	0.584	1.076	0.100	0.517	1.035	0.060	0.651	1.118	0.139
	cultivar 2	0.549	1.077	0.104	0.484	1.037	0.067	0.613	1.117	0.141
	cultivar 3	0.545	0.956	0.100	0.497	0.926	0.066	0.594	0.986	0.135
	cultivar 4	0.497	0.881	0.107	0.449	0.853	0.068	0.545	0.091	0.146
PCA	cultivar1	0.797	2.613	0.096	0.619	2.469	0.068	0.976	2.756	0.124
	cultivar 2	0.680	2.139	0.104	0.581	2.066	0.083	0.779	2.212	0.125
	cultivar 3	0.743	2.379	0.097	0.594	2.261	0.070	0.893	2.497	0.123
	cultivar 4	0.692	2.211	0.107	0.568	2.122	0.081	0.816	2.300	0.133
PH	cultivar1	0.338	2.630	0.150	0.041	2.387	0.076	0.636	2.874	0.225
	cultivar 2	0.289	2.091	0.139	0.123	1.955	0.093	0.454	2.228	0.185
	cultivar 3	0.332	2.050	0.133	0.134	1.893	0.080	0.530	2.207	0.185
	cultivar 4	0.254	1.525	0.170	0.107	1.427	0.092	0.401	1.623	0.248
